# Health Literacy among Filipino Domestic Workers in Macao

**DOI:** 10.3390/healthcare9111449

**Published:** 2021-10-27

**Authors:** Pak-Leng Cheong, Hui Wang, Wan Cheong, Mei Ieng Lam

**Affiliations:** Education Department, Kiang Wu Nursing College of Macau, Macau 999078, China; emmiewang@kwnc.edu.mo (H.W.); ch_wan@kwnc.edu.mo (W.C.); minalam@kwnc.edu.mo (M.I.L.)

**Keywords:** filipino, domestic workers, health literacy

## Abstract

Migrant worker is a global phenomenon that is associated with the health of individuals and populations. Filipino workers constitute the largest group of non-Chinese migrant workers in Macao, they are mainly employed as domestic workers. The purpose of this study is to investigate the status of health literacy (HL) and associated factors among Filipino domestic workers in Macao. The study is a cross-sectional study. Chi square’s test and binary logistic regression models were used for data analyzing. Filipino who was employed by a family in Macao as a domestic worker within the valid contract period was eligible in the study. A total of 379 valid questionnaires were collected during December 2020 and March 2021. Health literacy was measured using the short-form Health Literacy Instrument (HLS-SF12). The results showed that only 37.4% of the respondents have sufficient health literacy. Age was an important factor that was associated with health literacy, with Filipino domestic workers younger than and equal to 30 years of age more likely to have inadequate health literacy. The results will help to make recommendations for further research and public health policy.

## 1. Introduction

Rapid urbanization has led to the continuous growth of the migrant population globally. More than half of this population comprises migrant workers, who are persons moving from one country to another for employment [1]. According to the International Labour Organization (ILO) estimates, there were approximately 11.5 million migrant domestic workers worldwide. Almost 80 per cent of them were found in high-income countries that were experiencing demographic aging of societies and transformation of family structures, the demands are obvious [2].

Being a migrant worker is a global phenomenon that is associated with the health of individuals and populations. An increasing number of migrants are motivated by the desire to obtain low-skilled labor opportunities. With the globalization of population movement, migrant workers’ protection and public health policies need to be considered holistically and across multiple stages [3]. For example, migrant workers who enter a new region with their health beliefs also return to their place of origin. Going back-and-forth may be linked not only to the problem of infectious diseases but also to non-communicable diseases. This is because living conditions of most migrant workers increase health vulnerabilities, such as lack of family support, language and cultural differences, and inadequate social security. Furthermore, this makes it difficult to bear excessive costs, thereby affecting the workers’ health situation [4,5]. The World Health Organization (WHO) proposed developing public policies for migrant workers. It also emphasized promoting personal skills and health literacy, pointing out that health literacy was related to health status and the use of health facilities (such as medical compliance and participation in prevention activities) [6].

### 1.1. Health Literacy and Its Associated Factors

While many definitions for health literacy exist, the Institute of Medicine (US) defined health literacy as “everyone’s involvement in health promotion and protection, disease prevention and early screening, health care and maintenance, and policy making” [7]. Another definition was concluded by Kwan, Frankish, and Rootman, health literacy was the extent to which people were capable to obtain, understand, evaluate, and apply information to meet the needs of different health conditions, in order to facilitate and maintain good health throughout their lives [8]. For a comprehensive understanding of health literacy, Sørensen et al. conducted a systematic review and content analysis, and found that health literacy was closely related to people’s cultural level. People need to acquire, understand, evaluate, and apply health-related information based on existing knowledge, motivation, and ability so that they can make decisions related to medical care, disease prevention, and health promotion in daily life [9]. Health literacy can further influence people’s health behaviors and the use and equity of health services, thereby affecting the overall health outcomes and economic burden of the society [9,10].

Regarding the factors affecting health literacy, a survey in Spain of the general population aged 15 to 98 years (*N* = 2433) found that 15.4% showed insufficient or problematic health literacy, which was associated with lower educational level and lower socioeconomic status [11]. Another study in the Philippines surveyed adults from low-income families (*N* = 2036), and found that 93.8% of them had inadequate or problematic levels of health literacy and that a low level of education was a significant contributor to health illiteracy [12]. Thus, one may infer that people living with different levels of social development have different levels of health literacy.

Many studies have confirmed that demographic information has an important relationship with health literacy. A large survey of rural areas in mainland China (*N* = 1164) showed that age, body mass index (BMI), distance between residence and the nearest medical institution, monthly income, occupation, and education levels were significantly associated with health literacy; moreover, these factors could explain 70.2% of the total variation in health literacy [13]. These results are similar to those reported in other national studies. Using survey data from the Netherlands and Europe, Heide et al. also showed that age, gender, education level, monthly household income, and working status were important predictors of health literacy [14].

Language comprehension is also important for health literacy. A survey of Hispanic immigrants in the United States showed that English proficiency was the most important predictor of health literacy [15]. A national survey in Iran also showed that literacy is closely related to health literacy, which may be a confounding factor for high health literacy, such as high education levels and regular workers [16]. Briones, Palatino, and Agosto pointed out that despite the large amount of health information available to the public, the level of health literacy was still inadequate, reflecting the need to further evaluate whether health information is accessible, understandable, readable, and applied to make health decisions for individuals [12]. Filipino domestic workers in Macao have also reported that language issues are an important factor, besides economic and cultural factors, that impede access to medical services [17]. Therefore, information comprehension ability, especially language proficiency, may be an important factor associated with health literacy.

### 1.2. Filipino Domestic Workers

Domestic workers are an important source of employment for Filipinos, especially women, with about a quarter of the Filipinos workers abroad being domestic workers each year [18]. These workers were mainly destined for Asia [19], and their numbers have risen sharply in the past two decades. In Hong Kong and Singapore, for example, the number of Filipinos domestic workers rose from 22,000 in 2002 to 207,000 in 2020, and from 1428 in 2002 to 72,000 in 2015, respectively [18,20,21]. With its social and economic development, Macau has also become a destination of choice for Filipino domestic workers in recent years. Until 2019, the number of migrant employees in Macao was 196,100, accounting for approximately half (50.6%) of the labor population. Filipino workers constitute the largest group of non-Chinese migrant workers in Macao, were mainly employed as domestic workers, and number approximately 16,800 [22].

For domestic workers, there are no specific qualifications or skills required for entry. Domestic workers generally engage in cooking, cleaning, and caring for children and the elderly. According to the ILO, many domestic workers around the world have poor working conditions, especially long working hours, lack of guaranteed salary, and pregnancy benefits [23]. Although the rights, obligations, and protection of non-local employees in Macao are protected by relevant laws, the working conditions and social determinants of health in this population are still unsatisfactory [24]. Filipino domestic workers in Macao not only need to adapt to cultural and language differences but also faced different living and working environments. Some studies have pointed out that Filipino workers who migrate to work in Macao faced many job-related pressures during the adjustment process, such as excessive supervision at work [25], they experienced mental health problems such as depression, anxiety, and addiction behavior (such as gambling and alcohol abuse); lived in poor conditions, such as lack of privacy, working overtime, and discrimination; bore the labor fee and pressure of remittance for the family financial; and had a lack of social support [17]. It cannot be denied that with the social and economic development of Macao, more and more Filipino domestic helpers are working in Macao. The above data show that they live and work in a Chinese community, a special environment and have a certain healthcare needs. However, their health status is often neglected.

Therefore, the purpose of this study is to investigate the status of health literacy and the factors associated with health literacy among Filipino domestic workers in Macao. According to the conceptual frameworks of Health Literacy developed by Sørensen et al. [9], the research hypothesis was addressed in this study: demographic data included gender, age, marital status, years of working in Macao, educational background, working hours, living status, average monthly income, and Chinese and English proficiency are associated with health literacy among Filipino domestic workers in Macao.

## 2. Materials and Methods

### 2.1. Design

This was a cross-sectional study, and the primary variables included demographic data and health literacy. Chi-square tests and binary logistic regression models were used for data analysis.

### 2.2. Sample

According to the Labor Bureau of the Government of Macao SAR, there were approximately 16,800 Filipino domestic workers in Macao [22]. For this study, the inclusion criteria were as follows: 1. Nationality is Filipino, and 2. A foreign employee who was employed by a family in Macao in domestic work within the valid contract period. The exclusion criteria were as follows: 1. Informally employed for household chores, such as probations and part-time; and 2. Holder of Macao residence identity card. For the sample size, the allowable error was 5%, and the confidence was 1 − α = 0.95. The minimum sample size to be investigated was 375. Convenience sampling was used while cooperating with the five Filipino organizations, including church and non-governmental organizations, to contact research participants.

### 2.3. Data Collection

In order to ensure the quality of data collection, before starting this investigation, 20 college students were recruited and trained as investigators. Trained investigators contracted the eligible research respondents in cooperating organizations after obtaining permission from them. Survey monkey, an electronic questionnaire platform was used to collect the data. The first page of the electronic questionnaire provided informed consent only after the respondents agreed to participate in the electronic questionnaire.

### 2.4. Measurements

1. The demographic data included gender, age, marital status, years of working in Macao, educational background, working hours, living status, average monthly income, and Chinese and English proficiency.
2. The short-form Health Literacy Instrument (HLS-SF12) developed by Duong et al. [26] was adopted. This scale is a short version of the Health Literacy Questionnaire for the European population created by Sørensen et al. [9]. The scale contains three dimensions: Healthcare Literacy (HC-HL), which refers to the ability to obtain and understand medical information to make informed decisions and implement medical advice; Disease Prevention (DP-HL), the ability to obtain and understand information about health risks and make informed decisions to prevent disease; and Health promotion (HP-HL), the ability to regularly understand the determinants of health in social and living environments, and to make informed decisions and participate. There were 12 questions on this scale. Each question was scored on a 4-point Likert scale ranging from 1 = very difficult to 4 = very easy. The scale has been verified and appropriate for application to the general population in six Asian regions [26]. In this study, HLS-SF12 had undergone multiple forward translation from English to Filipino, two versions were translated respectively by two Filipinos whose second language was English. Then two Filipino domestic workers checked the words and phrases, and cultural appropriateness. Research team with a Filipino nurse then selected the most linguistically appropriate translation to produce the target instrument after discussing the domestic helper’s comments. For multiple back translation, two versions were translated respectively by two linguistics graduated students, then reviewed by a linguistics professor. Research team with a linguistics professor discussed this back translation. Monolingual test was implemented by two native English speakers, and bilingual test was also performed by two bilingual Filipino domestic helpers, for checking the equivalence of the original English version and translated English version, in order to ensure that the tool was suitable for the target group to investigate the health literacy status of Filipino domestic workers in Macao. Cronbach’s alphas for general health literacy and the three health literacy domains ranged from 0.83 to 0.94. Following the European Health Literacy Project, those with 33 or less HL index were defined as Limited HL [27].

### 2.5. Ethical Considerations

This study was approved by the Kiang Wu Nursing College of Macau (2020JAN01). Permission was obtained from all cooperative organizations. Written informed consent was obtained from respondents prior to starting the electronic questionnaire. All data were anonymous and only accessed by members of the research team.

### 2.6. Analysis Strategy

Statistical analysis was performed using SPSS version 26.0. Descriptive statistics included the mean, standard deviation, frequency, and percentage of each variable to understand the current situation of demography and health literacy of Filipino domestic workers in Macao. Inferential statistics included the Chi-square test and binary logistic regression, which were used to understand differences in health literacy among respondents with different demographic backgrounds.

## 3. Results

### 3.1. Respondents’ Characteristics

A total of 436 questionnaires were collected, of which 379 (86.9%) were effective. Invalid questionnaires mainly refer to incomplete questionnaires or not the target population of this study. 97.1% of the respondents were female, with an average age of 43.7 (±9.33) years. The average number of working years in Macao is 6.1 (±4.85) years, the average number of working hours per week is 68.6 (±15.81) hours, and the average monthly salary is MOP 4357.0 (±716.72). For other information, refer to Table 1. 

### 3.2. Respondents’ Health Literacy

The mean item score in this study was 2.5–2.9. A score of 1–2 was classified as difficult and 3–4 as easy. The distribution was shown in Table 2. The most difficult item for respondents was “judge which vaccinations you may need?”. The item most respondents found easy was “understand information in the media (such as Internet, newspaper, magazines) on how to get healthier?”. The General HL of respondents was 28.4 ± 8.6, Healthcare was 28.0 ± 9.4, Disease prevention was 27.0 ± 9.5, Health promotion was 30.2 ± 8.9, all of which belonged to the limited HL group. Only 37.4% respondents had sufficient General HL, 50.7% of the respondents had sufficient Healthcare HL, and 45.1% and 59.6% of the respondents had sufficient Disease prevention HL and Health promotion HL, respectively. For more details, refer to Table 2.

### 3.3. The Association between Health Literacy and Demographic Characteristics

A chi-square test was performed to examine the relationship between health literacy and demographic characteristics. Respondents who were older 30 years old and with good English proficiency were more likely to have adequate general HL (X^2^ = 7.83, *p* < 0.05; X^2^ = 9.05, *p* < 0.05). Women with good or fair English proficiency were more likely to have adequate Healthcare HL (X^2^ = 4.78, *p* < 0.05; X^2^ = 13.66, *p* < 0.01). Respondents with good English proficiency were more likely to have adequate Disease prevention HL (X^2^ = 14.67, *p* < 0.01). Female who were older than 30 years old, with good or fair English proficiency were more likely to have adequate Health promotion HL (X^2^ = 8.09, *p* < 0.01; X^2^ = 14.20, *p* < 0.01; X^2^ = 12.10, *p* < 0.01). For more details, refer to Table 1.

In binary logistic regression analysis, indexes of General HL, Healthcare HL, Disease prevention HL, and Health promotion HL were used as binary variables, respectively. Indexes greater than (less than or equal to) 33 were considered as sufficient (limited) health literacy. All factors associating health literacy were included as independent variables. The results show that only age in General HL and Health promotion HL were statistically significant. Older people are less likely to have limited General HL and Health promotion HL. Respondents aged 31 to 42 years (OR = 2.820, *p* < 0.05) were 1.8 times more likely to have adequate General HL than those younger than and equal to 30 year. Respondents aged older than 30 years (OR = 2.836–3.019, *p* < 0.05), were 1.8–2.0 times more likely to have adequate Health promotion HL than those younger than and equal to 30 years. For more details, refer to Table 3.

## 4. Discussions

This study showed that more than half of Filipino domestic workers have inadequate HL in Macao and the factors that are associated with the health literacy include gender, age, and English proficiency. To the best of our knowledge, this is the first survey of the health literacy of Filipino domestic workers in Macao.

### 4.1. Poor Working and Living Conditions

Unsurprisingly, most respondents in this study were women. This was because domestic workers were highly feminized careers, 80% of all domestic workers are women worldwide [28]. And we found that the respondents in this study were facing poor working and living conditions, such as long working hours, low wages, limited language, and lack of social support. These unsatisfactory social determinants of heath were also faced by domestic workers around the world, further affecting their health and quality of life [23,24].

### 4.2. Low Health Literacy

We found that the General HL index of Filipino domestic workers was 28.4 ± 8.6. Only 37.4% respondents had sufficient General HL. This means that most of this population was not capable of accessing, understanding, evaluating, and communicating to meet the needs of different health situations. Compared with six Asian regions, the General HL of the study group was lower than that of Myanmar (29.2 ± 9.3), Vietnam (29.5 ± 9.5), Indonesia (30.5 ± 6.4), Kazakhstan (31.6 ± 9.5), Malaysia (32.7 ± 7.9), and Taiwan (34.3 ± 6.9) [26]. Notably, migrant workers living in unfamiliar places are affected by a variety of factors and their level of health literacy may not be comparable to that of the general population living in their country. The limited evidence generally reports that migrant workers have relatively low levels of health literacy [29,30]. This is consistent with the results of this study.

Moreover, this study found that among the 12 questions about health literacy, the average score of all items did not reach the “easy” level, and the three dimensions of the scale, including Health care, Disease prevention, and Health promotion, did not reach the “sufficient” level. This shows that the overall health literacy of the study respondents is insufficient. Although few studies have analyzed the performance of each item and each dimension, it cannot be denied that the health literacy of the respondents in this study needs to be paid attention to and strengthened. For example, the most difficult item for respondents was “judge which vaccinations you may need?”. Notably, this study was conducted in the context of COVID-19, and respondents’ consideration of vaccination needs was different from usual, reflecting the limited access and application of relevant information by this group.

### 4.3. Factors Associated with Health Literacy

The study found the following factors to be associated with health literacy: gender, age, and language proficiency. Gender has been consistently noted by many studies, women generally have better health literacy [12,13,14]. Since great majority domestic workers are female [28], this study is no exception. Regarding the correlation between gender and health literacy, this study was limited by a small number of male respondents, which might require further discussion.

Age was found to be an important factor associated with health literacy. Filipino domestic workers aged greater than 30 years were more likely to have adequate health literacy than those younger than and equal to 30 years. Many studies have found that people with younger age have better health literacy [13,14], which seems different to the results of this study. However, the respondents of this study were labor force, age between 19 to 66 years, without the elderly and minors; thus, the results of this study are relatively similar to the results of other studies, age is an important factor associating health literacy and most middle-aged adults have better health literacy. Therefore, the health needs of Filipino domestic workers under and equal to the age of 30 are often overlooked. This study found that the health literacy of Filipino domestic workers under and equal to the age of 30 was inadequate and needs to be addressed.

Many studies have found that language proficiency had an important impact on health literacy, familiarity with the local language particularly [15,17,31]. Chinese is an official language in Macao, this study found that most Filipino domestic workers did not have a good level of Chinese. Interestingly, English proficiency was more influential than Chinese proficiency for their health literacy. Most respondents in this study had a fundamental education level and English proficiency, which was related to their background before migrating to Macao. Some studies had explored the effects of language courses on the health literacy of migrant workers. Although language courses can help partially promote health literacy, it was more important to provide environmental and social support to promote health [32]. Therefore, Filipino domestic workers with low English proficiency need more attention and support so that accessible and understandable health information can be provided to them to apply and make health decisions.

### 4.4. Implications

The health needs of Filipino domestic workers have received less attention because of their unique status, increasing their health vulnerability. This study found that the health literacy of this group was insufficient, which might have a negative impact on their health. It is suggested to pay more attention to the health needs of this group, provide more accessible and readable health information, and support them to apply this information to make health decisions. Although this study was conducted in Macao, the results are of certain value to the cities with similar backgrounds and employ many Filipino domestic workers, such as Hong Kong and Singapore. Moreover, it is suggested that to further understand the Filipino domestic workers in the acquiring, understanding, evaluation, and application of health-related information. For example, qualitative studies or mixed methods study explore how health systems can provide a friendly environment and social support for this group to promote their health.

### 4.5. Limitations

A cross-sectional design was adopted in this study, the respondents reported their condition subjectively, it was difficult to assess their actual status. Besides, the respondents of this study have special backgrounds, they immigrated to Macao from Philippines, and this study measured their conditions when they lived in Macao, not including their conditions in the Philippines, and the results were only local conditions. Moreover, this study sample were recruited through cooperation organizations, most of them are religious organizations. Thus those who did not participate in the cooperation organization, we failed to cover.

## 5. Conclusions

Migrant workers are one of the groups engaged in the phenomenon of global population migration. They leave the place of origin and moved to the destination to work. Environmental changes affect the life and health status of the group. Recently, the health of migrant workers has become a focus of global public health. This study investigated the status of health literacy and the factors associated with health literacy among Filipino domestic workers in Macao. We found that their health literacy was insufficient. The factors associated with their health literacy included gender, age, and language proficiency. Age was an important factor and Filipino domestic workers younger than and equal to 30 years of age were more likely to have poor health literacy. These results may be helpful for determining future research directions and enacting public health policies.

## Figures and Tables

**Table 1:** Demographic Characteristics of respondents and the association to health literacy (*N* = 379).

Characteristics		General HL			Healthcare HL			Disease Prevention HL			Health Promotion HL		
	*N* (%)	>33	≤33	X^2^	>33	≤33	X^2^	>33	≤33	X^2^	>33	≤33	X^2^
**Gender**				1.75			4.78 *			3.32			8.09 **
1. Male	11 (2.9)	2	9		2	9	(1 < 2)	2	9		2	9	(1 < 2)
2. Female	368 (97.1)	141	229		190	178		169	199		224	144	
**Age (years)**				7.83 *			3.98			4.50			14.20 **
1. 19–30	34 (9.0)	6	28	(1 < 2)	12	22		10	24		10	24	(1 < 2&3&4)
2. 31–42	114 (30.1)	50	64		62	52		57	57		72	42	
3. 43–54	188 (49.6)	70	118		95	93		85	103		117	71	
4. 55–66	43 (11.3)	15	28		23	20		19	24		27	16	
**Marital status**				2.83			0.17			0.88			1.24
1. Single	136 (35.9)	43	93		67	69		57	79		76	60	
2. Married	243 (64.1)	98	145		125	118		114	129		150	93	
**Years of working in Macao**				3.12			0.82			1.05			0.26
1. <6 years	229 (60.4)	70	101		91	80		80	91		101	70	
2. 6–10 years	96 (25.3)	56	98		75	79		70	84		94	60	
3. ≥11years	54 (14.2)	15	39		26	28		21	33		31	23	
**Educational background**				0.03			1.14			0.47			0.44
1. Secondary school or below	178 (47.0)	67	111		85	93		77	101		103	75	
2. College and above	201 (53.0)	74	127		107	94		94	107		123	78	
**Working hours weekly**				2.19			7.84			1.11			4.78
1. ≤48 h	73 (19.3)	22	51		29	44		30	43		36	37	
2. 49–60 h	61 (16.1)	22	39		32	29		28	33		39	22	
3. 61–72 h	159 (42.0)	63	96		92	67		76	83		101	58	
4. >72 h	86 (22.7)	34	52		39	47		37	49		50	36	
**Living status**				3.59			5.56			0.77			3.05
1. Living with employer	219 (57.8)	87	132		112	107		99	120		132	87	
2. Living with family	38 (10.0)	9	29		13	25		15	23		18	20	
3. Living with friends	101 (26.6)	37	64		57	44		48	53		64	37	
4. Living alone	21 (5.5)	8	13		10	11		9	12		12	9	
**Monthly income**				6.58			3.34			7.20			4.16
1. ≤ MOP 4000	139 (36.7)	46	93		65	74		57	82		80	59	
2. MOP 4001–4500	150 (39.6)	67	83		84	66		80	70		95	55	
3. MOP 4501–5000	66 (17.4)	19	47		33	33		24	42		34	32	
**English proficiency**				9.05 *			13.66 **			14.67 **			12.10 **
1. Good	250 (66.0)	103	147	(3 < 1)	138	112	(3 < 1&2)	127	123	(2&3 < 1)	158	92	(3 < 1&2)
2. Fair	119 (31.4)	38	81		54	65		44	75		67	52	
3. Poor	10 (2.6)	0	10		0	10		0	10		1	9	
**Chinese proficiency**				3.63			2.84			2.01			4.74
1. Good	6 (1.6)	0	6		1	5		1	5		1	5	
2. Fair	36 (9.5)	14	22		18	18		16	20		21	15	
3. Poor	337 (88.9)	127	210		173	164		154	183		204	133	

* *p* < 0.05; ** *p* < 0.01.

**Table 2:** Score of HLS-SF12 (*N* = 379).

Health Literacy Questions		Mean (SD)	Difficulty n(%)	Easy n(%)
1	To find information on treatments of illnesses that concern you?	2.7 (0.7)	117 (30.9)	262 (69.1)
2	Understand the leaflets that come with your medicine?	2.7 (0.7)	104 (27.4)	275 (72.6)
3	Judge the advantages and disadvantages of different treatment options?	2.6 (0.7)	156 (41.2)	223 (58.8)
4	Call an ambulance in an emergency?	2.8 (0.7)	87 (23.0)	292 (77.0)
5	Find information on how to manage mental health problems like stress or depression?	2.6 (0.7)	135 (35.6)	244 (64.4)
6	Understand why you need health screenings (such as breast exam, blood sugar test, blood pressure)?	2.7 (0.7)	109 (28.8)	270 (71.2)
7	Judge which vaccinations you may need?	2.5 (0.7)	170 (44.9)	209 (55.1)
8	Decide how you can protect yourself from illness based on advice from family and friends?	2.7 (0.7)	119 (31.4)	260 (68.6)
9	Find out about activities (such as meditation, exercise, walking, Pilates etc.) that are good for your mental well-being?	2.8 (0.6)	75 (19.8)	304 (80.2)
10	Understand information in the media (such as Internet, newspaper, magazines) on how to get healthier?	2.9 (0.7)	74 (19.5)	305 (80.5)
11	Judge which everyday behavior (such as drinking and eating habits, exercise etc.) is related to your health?	2.8 (0.6)	87 (23.0)	292 (77.0)
12	Join a sports club or exercise class if you want to?	2.7 (0.7)	105 (27.7)	274 (72.3)
Health literacy domains		Mean of HL index (SD)	Limited HL n(%)	Sufficient HL n(%)
Health Care (items 1–4)		28.0 (9.4)	187 (49.3)	192 (50.7)
Disease Prevention (items 5–8)		27.0 (9.5)	208 (54.9)	171 (45.1)
Health Promotion (items 9–12)		30.2 (8.9)	153 (40.4)	226 (59.6)
General HL		28.4 (8.6)	238 (62.6)	141 (37.4)

**Table 3:** Binary logistic regression analysis in Health promotion HL (*N* = 379).

	Age (Years)	B	Wald	OR	*p*	95% CI	
General HL	31–42 vs. ≤30	1.037	4.303	2.820	0.038	1.059	7.513
	43–54 vs. ≤30	0.736	2.274	2.087	0.132	0.803	5.429
	≥55 vs. ≤30	0.626	1.236	1.870	0.266	0.620	5.642
Health Promotion HL	31–42 vs. ≤30	1.105	6.097	3.019	0.014	1.256	7.256
	43–54 vs. ≤30	1.043	5.756	2.836	0.016	1.210	6.648
	≥55 vs. ≤30	1.048	4.130	2.851	0.042	1.038	7.831

## Data Availability

All data that support the findings of this study are available from the corresponding author upon reasonable request.
